# Book Reviews

**Published:** 1899-04

**Authors:** 


### Book Reviews.

A TEXT-BOOK OF OBSTETRICS. By BARTON COOKE HIRST, M. D., Professor of Obstetrics in the University of Pennsylvania. With 653 illustrations. Octavo, pp. 846. Philadelphia: W. B. Saunders. 1898.

There have been a good many works on obstetrics issued within the last ten years, most of which possess excellence in one or more particulars, and all have added immensely to the valuable and, let us hope, permanent literature of the subject. In view of this fact a new treatise, that aims to be a text-book, must present some special claims in order to arrest attention or to merit thoughtful consideration. It is gratifying to discover early in its examination such a book, for it makes the reviewer's task a pleasant one, because it is always an irksome duty to review an unnecessary, not to say a useless, addition to our already overstocked book market.

The author begins his work with the consideration of pregnancy to which the first part, consisting of seven chapters, is devoted. Anatomy naturally occupies first place and the subject is most admirably presented and illustrated. More attention should be given by students to the study of the bony pelvis than is usually the case, and they should also thoroughly acquaint themselves with the pelvis in its clothed state. This book will be most helpful in this direction. The second chapter considers menstruation, ovulation and fertilisation. The development of the embryo and fetus, which the third chapter describes is a very interesting part of the book. The fetal appendages are considered in the fourth chapter and in the fifth the diseases of the fetus are dealt with, in methods that merit most favorable commendation.

The physiology of pregnancy always possesses absorbing interest to the student of the obstetric art, and it should be carefully studied and thoroughly mastered by every one who intends to practise obstetrics. The chapter that Hirst devotes to this part of the subject is a well-written exposition of it, and cannot fail to interest as well as instruct the reader. It contains the most modern thought on the essentials connected with the topic and is especially strong in the diagnosis of pregnancy. Following next is a remarkably interesting chapter on the pathology of the pregnant woman, which takes the student or reader from the initial subdivision, diseases of the genitalia, along through diseases of the uterine muscle, of the alimentary canal, of the urinary apparatus, of the nervous system, of the circulatory system, of the respiratory apparatus, of the osseous system, of infection, and of the skin, until finally are considered abortion, miscarriage, premature labor, and lastly, extrauterine pregnancy. The author adheres to the ordinary classification of premature expulsion of the ovum and fetus, terming it *abortion* if before the fourth month, *premature labor* after viability, and *miscarriage* if between these two periods. This is so simple, and so easily understood, even by the tyro, that we wish teachers would universally adopt it. There are some things that all may agree upon and it seems to us this is one of them.

In the second part of the book we find the consideration of labor begun, its physiology and management and the management of the puerperium taking up this part; then in the third part the mechanism of labor is fully described in all its details; in the fourth part the pathology of labor is dealt with and in the fifth the pathology of the puerperium is explained. Obstetric operations, from the induction of abortion to Cesarean section, occupy part six, and the seventh and last part, is devoted to the new-born infant, its physiology, pathology injuries and diseases. Every one of these several parts bear the impress of the master, each and all indicating a personal experience by the writer in the lecture-room and in the clinic. The only way to teach practical obstetrics is at the bedside, and Hirst is recognised as an obstetrician of great experience in the lying-in room. Every student before graduation should have the opportunity to attend several cases of labor, and the seniors should conduct for themselves at least six confinements without interference on the part of the instructor, except to be within call if needed. This would give the undergraduate a sense of personal responsibility that could not help make him self-reliant in his earlier obstetric practice.

Such a book as this one makes the literature of medicine better and is a credit to American letters. It is a safe guide for the practitioner and contains the essentials of the art of obstetrics up to the moment of its issue from the press. Its illustrations are far above the common, most of them being original, and they make plain many a subject that is intricate to the tyro. It is well named a textbook and should so become in every medical school of standing.

Practical Diagnosis. The Use of Symptoms in the Diagnosis of Disease. By Hobart Amory Hare, M. D., Professor of Therapeutics and Materia Medica in the Jefferson Medical College of Philadelphia. Third edition, enlarged and thoroughly revised. In one 8vo volume of 615 pages, with 204 engravings and 13 full-page covered plates. Philadelphia and New York: Lea Brothers & Co. 1898.

It is somewhat remarkable, in view of the many books on this subject or bearing close kinship to it, that Prof. Hare's treatise should have advanced to its third edition in two years after its first appearance. This is the almost universal thought at first glance—this is "snap diagnosis." But a little closer examination of the work will disclose ample reason for its popularity and explain why it has, in a measure, outstepped others in the race for professionl and especially for text-book favor.

We have heretofore in previous notices of the book, called attention to the method Dr. Hare adopts in arranging the subject—one that is unique and withall, most useful and instructive. By making the symptoms units and arranging them under two general heads—the manifestation of disease in organs, and the manifestation of disease by symptoms—he attains simplicity in the presentation of the subject and commands at once attention to its salient features. The fact that these two general subdivisions are placed in reverse order, is an additional reason why the treatise meets favor, for this is after all the logical way to deal with diagnostic questions.

This edition has been thoroughly revised, bringing it forward to the immediate present as a guide to diagnostic precision. The rapid strides of medical science make it impossible for any work to keep pace with it, unless through the medium of frequent additions, hence it is better to choose such a book when a choice must be made between several. The present edition retains all the features of its predecessors and much new material has been added. It is well indexed, generously and carefully illustrated, and altogether is the most satisfactory work on diagnosis extant. This and Hare's Practical Therapeutics make a pair that it is difficult to match and still more difficult to beat.

A Treatise on Diseases of the Ear; together with a Brief Sketch of the Anatomy and Physiology of this Organ By Albert H. Buck, M. D., Clinical Professor of the Diseases of the Ear, College of Physicians and Surgeons, Medical Department of Columbia University, New York; Consulting Aural Surgeon, New York Eye and Ear Infirmary and the Presbyterian Hospital. Third revised edition. Octavo, pp. xii.—592. New York: William Wood & Co. 1898.

This treatise has been familiar to the profession through its previous editions and it has been already accepted by otologists as a standard work on the ear. The author has had a very large experience in this branch of medical science and he is a most attractive writer. His large acquaintance with the medical world, made as the editor of Ziemssen's Cyclopedia of Practice, gives him the confidence of physicians everywhere.

The field now covered by the oral surgeon is an extensive one, having been very much enlarged during recent years. First the nasal tract was added and now many intracranial diseases of otitic origin have been annexed, thus very materially increasing the responsibilities of the otologist. The author of this work has constructed it on the broadened domain here indicated. The text of the treatise has been fully and completely revised, embracing everything written in the legitimate field of otology, according to its present boundary lines. This made necessary an entirely new set of plates, so that nothing might be left undone to modernise the book in accordance with the most advanced state of science. We regard it as a treatise not only useful to the specialist, but from which the general practitioner of medicine will derive great benefit.

